# Metabolic Response of Aphid *Cinara tujafilina* to Cold Stress

**DOI:** 10.3390/biology10121288

**Published:** 2021-12-07

**Authors:** Roma Durak, Tomasz Durak

**Affiliations:** Institute of Biology and Biotechnology, University of Rzeszów, Pigonia 1, 35-310 Rzeszów, Poland; tdurak@ur.edu.pl

**Keywords:** metabolic profile, amino acid, aphids, insect, overwintering

## Abstract

**Simple Summary:**

Aphid species that have widened their ranges due to climate change, like other insects, must adapt to low temperatures to avoid death. The metabolic changes in the body of the aphid that enabled it to survive the low temperatures of winter were investigated. Features, such as reducing their metabolic rate, an accumulation of sugars and polyols, and increased levels of some amino acids and fatty acids allow the aphids to overwinter as active-stage on the plant.

**Abstract:**

Climate changes enable thermophilic insect species to expand their ranges, but also force them to adapt to unfavourable environmental conditions in new habitats. Focusing on *Cinara tujafilina*, we investigated the metabolic changes in the body of the aphid that enabled it to survive the low temperatures of winter. Using GC–MS analysis, differences in the chemical composition of the aphids in summer and winter were found. The metabolic changes were mainly related to the increased activity of the pathways of carbohydrate metabolism, such as glycolysis and the pentose phosphate pathway; a decrease in tricarboxylic acid cycle (TCA); accumulation of polyols; and increased levels of proline, tyrosine, and fatty acids.

## 1. Introduction

Climate change allows insects to expand their ranges [1,2,3] but also forces them to develop adaptations that will enable them to survive uncomfortable environmental conditions, such as low temperatures in winter. Biochemical mechanisms protect the insect’s organism against cold stress by changing its metabolite concentrations [4,5]. Metabolic changes in the body of an insect indicate the main directions of its response to stress. The synthesis and accumulation of sugars and polyols (sugar alcohols) as cryoprotectants and some amino acids are the main processes observed in insects [6,7]. These substances protect cell membranes and enzymes against the effects of low temperatures [8]. Trehalose, glycerol, and myo-inositol have been shown to be the main cryoprotectants in insects [7,9,10]. Moreover, it was found that amino acids, such as Pro, Ala, and Leu, were responsive to cold stress [11,12,13].

Aphid species that have widened their ranges due to climate change, like other insects, must adapt to low temperatures to avoid death. This is especially true of species that reproduce by parthenogenesis throughout the whole year, omitting the egg stage. One of such species is aphid *Cinara tujafilina* (del Guercio, 1909) (Hemiptera, Lachninae), a native species of warm regions of Asia, which was introduced to the temperate regions of Europe [14]. This species adapted to overwintering by changing its settlement place from leaves where it fed in the summer to the root which it inhabited in winter [15]. Our previous studies have shown behavioural, morphological, developmental, and biochemical adaptations of this species that allow it to adapt to cold stress connected with overwintering, indicating the possibility of accumulation of cryoprotectants [15,16]. The aim of the current research was to determine metabolic changes that take place in the bodies of aphids which enable them to overwinter as active-stage on the plant.

## 2. Materials and Methods

### 2.1. Aphids and Sample Collection

*C. tujafilina* aphids came from cultures grown at the University of Rzeszow (Poland). Aphids were grown on *Thuja orientalis* plants under controlled conditions in climatic chambers (MLR-351H; Sanyo Corp., Osaka, Japan). Before starting the experiment, the plants with adult wingless females were kept at 20 °C to acclimatise for 2 weeks. During experiment, aphids were kept at a temperature of 20 ± 1 °C, humidity 60 ± 5%, and with a photoperiod of L/D = 16:8 to simulate summer conditions, and at a temperature of 5 ± 1 °C, humidity 60 ± 5%, and with a photoperiod of L/D = 8:16 to simulate winter conditions for 1 month. This range of temperatures was chosen because at temperatures below 10 °C, aphids of this species migrate to the stem and root of the plant where they develop a winter form [15]. We selected a summer temperature range that could be considered as typical in a temperate climate and is optimal for the development of most aphid species. Summer samples were collected from the leaves, and winter samples were collected from the main stem near the root collar or from the roots of the plant. Each sample contained 30 individuals of adult apterous females. Three independent samples were taken for each period.

### 2.2. Metabolomic Analysis

Sample preparation was done according to publication of Michaund and Denlinger [17]. A total of 100 µL of each sample was transferred to a conical glass vial prior to GC separation. Metabolites were identified using a GC–MS system (TRACE 1310 GC oven with TSQ8000 triplequad MS from Thermo Scientific, MA, USA) equipped with DB-5MS column (30 m × 0.25 mm × 0.25 µm) (J&W Scientific, Agilent Technologies, Palo Alto, CA, USA). Chromatographic separation conditions in gradient mode were kept as follows: 70 °C for 2 min, followed by 10 °C/min up to 300 °C, at 300 °C (10 min). PTV injector was used for sample injection with temperature gradient from 40 to 250 °C; column interface was kept at 250 °C and source temperature at 250 °C. Ion source operated in the *m/z* range: 50–850; in EI positive mode, electron energy was set to 70 eV.

Raw data files were converted to abf format for analysis using MSDial software (v. 3.96). To eliminate the retention time (Rt) shift and to determine the retention indexes (RI) for each compound, we implemented the correction against alkane series mixture (C-10 to C-36) directly in MS Dial. For compound identification, MSP database from CompMS site containing 28,220 records was used. Identified artifacts (alkanes, column bleed, plasticizers, MSTFA, and reagents) were excluded from further analyses. Obtained normalized (using total ion current (TIC) approach) results were then exported to Excel for pre-formatting and then used for statistical analyses.

### 2.3. Statistical Analysis

A *t*-test was performed, and metabolites with a *p*-value of < 0.05 were considered differential metabolites between the summer and winter groups of aphids. Principal component analysis (PCA) and hierarchical cluster analysis (HCA) were performed to show similarities between winter and summer samples. All statistical analyses were done using Statistica version 13 programme (TIBCO Software Inc., 2017, http://statistica.io; accessed on 22 October 2021) and PAST 4.0 software (Øyvind Hammer, Natural History Museum, University of Oslo, Oslo, Norway) [18]. 

## 3. Results

**Figure 1:**
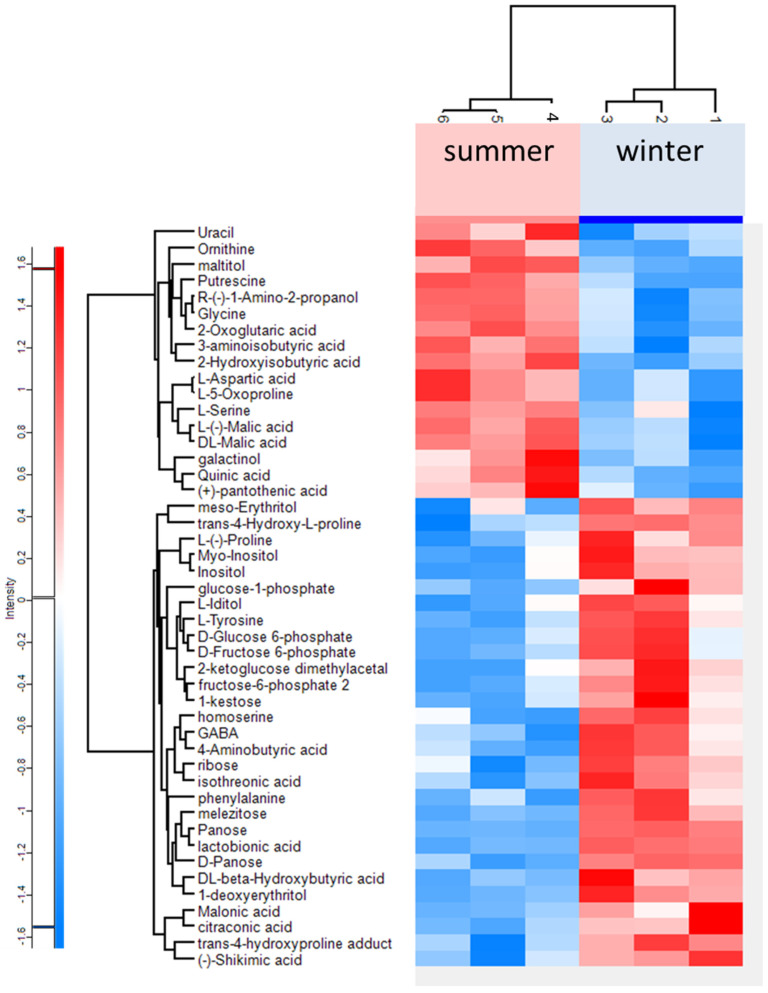
Heat map of metabolites identified in the *Cinara tujafilina* bodies from summer and winter samples; red colour indicates metabolites with a high expression, blue indicates metabolites with low expression.

GC–MS analysis identified 180 chemical compounds present in the body of aphids. Significant metabolic changes in the body composition of aphids in summer and winter were found in 46 chemical compounds, as shown in Figure 1. The main observed metabolic changes concerned the metabolites connected with energy metabolism; composition and level of carbohydrates and polyols, amino acids, and derivatives; and in organic acids.

Data from GC–MS analysis showed changes in primary metabolites linked to different metabolic processes. The increase was shown by compounds related to the glycolysis process and the pentose phosphate pathway (D-glucose 6-phosphate, glucose-1-phosphate, fructose 6-phosphate 2, D-fructose 6-phosphate, ribose) (Figure 1). The summer samples contained higher levels of malic acid and succinic acid. Winter samples contained higher levels of carbohydrates and polyols such as myo-inositol, inositol, iditol, erythritol, panose, kestose, and melezitose. In the winter samples, D-threitol, ribitol, trehalose, glycerol, and sorbitol were also found in small amounts, but always two- to fourfold higher than in the summer samples. An increase in organic acids was also observed in winter samples, such as shikimic, citraconic, malonic, and lactobionic (Figure 1). Winter samples contained higher levels of fatty acids. Among saturated acids, the highest increase was observed for stearic acid, which increased nearly sixfold, and for octanoic acid, the level of which increased more than twofold. Winter samples also contained an over threefold higher level of unsaturated fatty-oleic acids.

**Figure 2:**
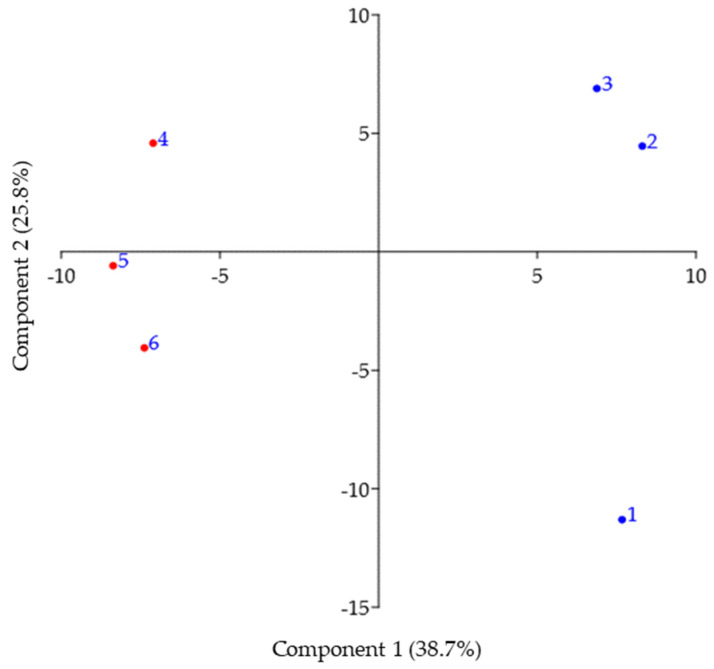
Principal component analysis (PCA) of metabolomic samples from winter (1, 2, 3) and summer (4, 5, 6) bodies of *Cinara tujafilina*.

Principal component analysis (PCA) of metabolomic samples from summer and winter samples of *C. tujafilina* are presented in Figure 2. The first two PCA axes covered 64.4% of the variance in the data. PCA analysis confirmed the metabolomic differences between the body of *C. tujafilina* in winter and summer (Figure 2).

**Figure 3:**
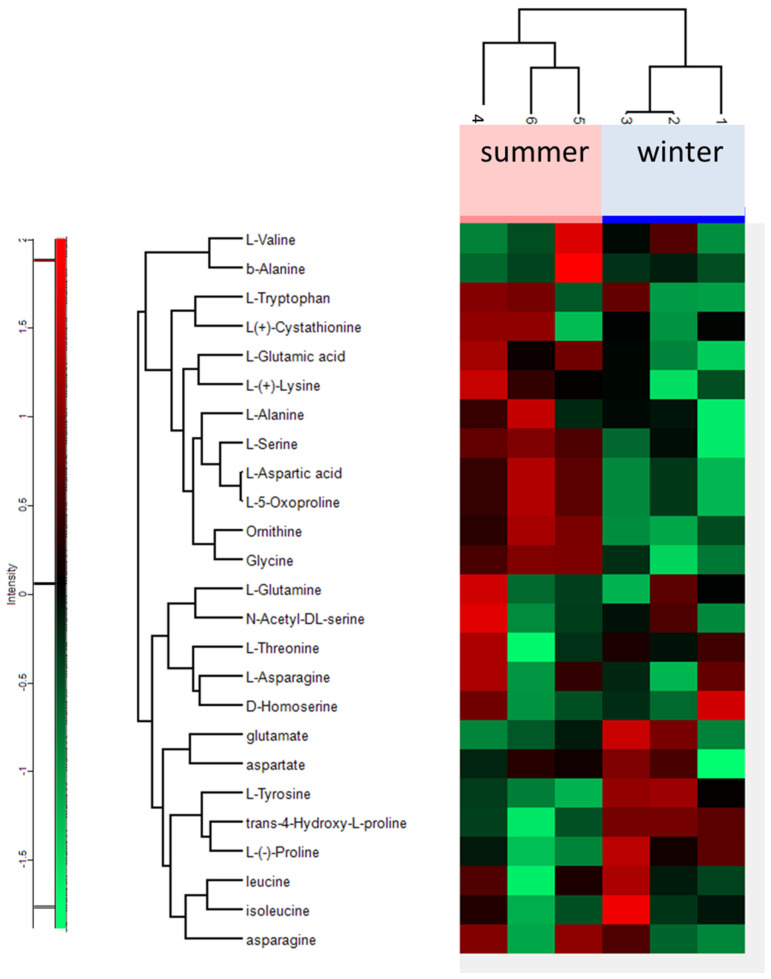
Heat map of amino acids and derivatives identified in the *Cinara tujafilina* bodies from summer and winter samples; red colour indicates metabolites with a high expression, green indicates metabolites with low expression.

Seventeen amino acids were detected in the aphid’s body. The total amount of free amino acids was slightly higher in summer samples than in winter samples (*p* > 0.05), but changes in the amount of individual amino acids were observed (Figure 3).

**Figure 4:**
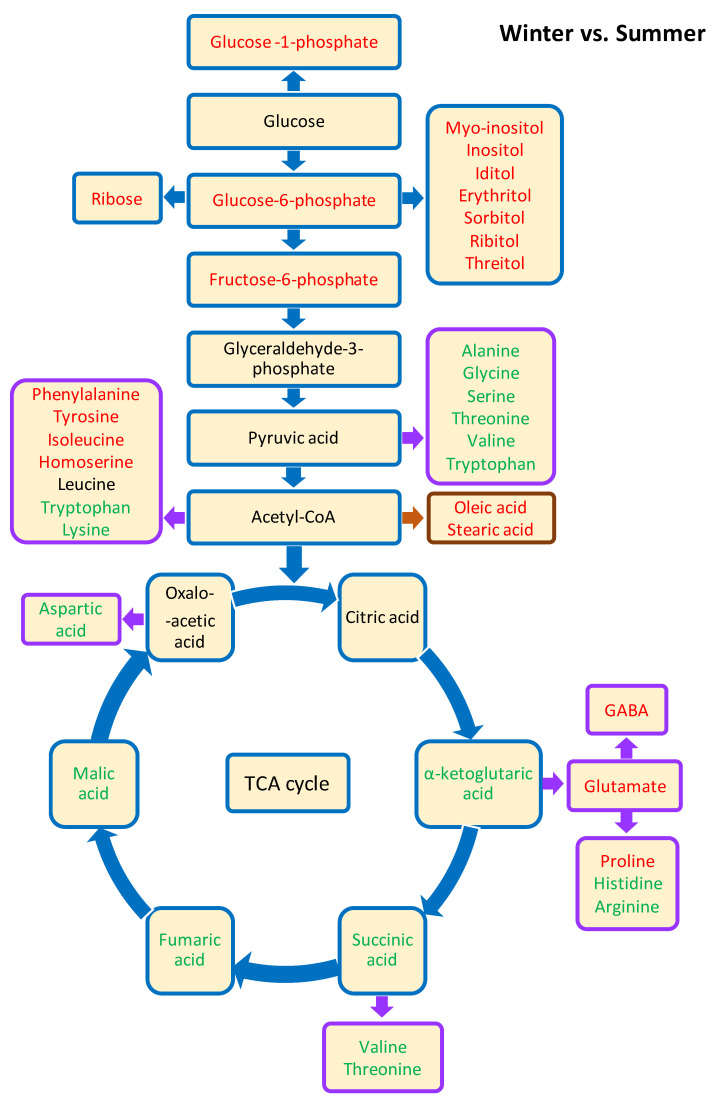
A simplified model of metabolite changes in aphids between the two distinct seasons. Blue arrows and boxes, carbohydrate metabolism; purple arrows and boxes, amino acid metabolism; brown arrow and box, fatty acid metabolism. Red colour indicates upregulated metabolites, green indicates downregulated metabolites, and black indicates no significant change in metabolite levels.

Hierarchical cluster analysis (HCA) analysis showed changes in the qualitative and quantitative composition of free amino acids that occur in aphid organisms during the winter (Figure 3). The number of amino acids and derivatives showing decreased levels was greater than the number showing increased values in the body of the overwintering aphids, compared with the summer insects. In wintering aphids, a significant increase mainly in tyrosine (Tyr) and proline (Pro,) but also in isoleucine (Iso) and phenylalanine (Phe), was found. Tyr and Pro accounted for as much as 42% of the total amount of free amino acids in aphids grown in winter conditions. A tenfold increase in Pro and a twofold increase in Tyr were found in the winter samples compared to the summer samples. The summer samples were rich in glycine (Gly), lysine (Lys), alanine (Ala), aspartic acid (Asp), and glutamic acid (Glu), the level of which was lower in the winter samples (Figure 3). A simplified model of metabolite changes in aphids between the two distinct seasons is shown in Figure 4. The analysis of the activity of the main metabolic pathways, such as the TCA cycle and pentose phosphate pathway, was performed on the basis of measuring the activity of intermediates such as malic acid or ribose.

## 4. Discussion

One of the main mechanisms of counteracting the effects of low temperature on an insect’s body is the activation of protective metabolic processes. The main pathways that are activated during cold stress are the carbohydrate metabolism pathways such as glycolysis, gluconeogenesis, and the pentose phosphate pathway, hence the increased level of sugars necessary for these processes, as observed in winter feeding aphids. Similar processes were also observed in *Sarcophaga bullata* [19,20]. In aphids subjected to cold stress, we observed in particular increased activity of the pentose phosphate pathway relative to the TCA cycle and accumulated polyols. A similar decrease in TCA cycle and respiratory chain activity was observed in a study of the aphid *Macrosiphum euphorbiae* after heat and radiation stress [21]. The decrease in the intermediates in TCA cycle, such as malic acid and succinic acid in aphids, suggests a shift from glycolysis to the pentose phosphate pathway, providing NADPH for sugar alcohol production [10,22].

The increase synthesis of sugars and polyols, which have the effect of cryoprotectants, was observed in the tissues of many species of insects. The most common metabolites with protective properties are the fructose and trehalose and polyols: glycerol, sorbitol, erythritol, and threitol [10,23]. The accumulation of these compounds increases the osmotic potential of the fluids of the aphid body, which prevents freezing. In addition, the accumulation of sugars such as panose, kestose, melibiose, and melezitose by wintering aphids protects the processes of obtaining energy from sugars by these insects in winter. High levels of polyols have been found in other insects subjected to cold stress, e.g., beetles [8,23] and moths [24]. The analysis of sugars and polyols of the aphids *Bravicoryne brassicae, Schizaphis graminum, Diuraphis noxia*, and *Aphis gossypii* mainly revealed in their body the presence of mannitol, trehalose, and myo-inositol [25,26,27]. The process of cryoprotectant accumulation in *C. tujafilina* showed an increase of five compounds (glucose, mannitol, trehalose, myo-inositol, glycerol) and a decrease in fructose levels [16]. Our current results showed a wider range of aphid substances that can be cryoprotectants that protect the insect’s body during wintering. Osmolytes such as threitol, erythritol, iditol, ribitol, and sorbitol are commonly found in insects as cryoprotectants [4,23], but thus far have not been found in aphids. A characteristic feature of these compounds is that they fulfil their function as cryoprotectant, even if their amount in the insect’s organism is a trace amount.

Our research showed the total amount of free amino acids was slightly higher in summer samples than winter samples (statistically insignificant), but significant changes were observed in the amount of individual amino acids. Similar results were obtained in studies of beetles in which the level of free amino acids decreased as the temperature decreased [11,13,28], but some studies indicated an increase in free amino acids in insects subjected to cold stress [12,29]. In the bodies of wintering aphids, a significant increase in tyrosine and proline was demonstrated. Tyrosine is usually available to insects in their food, and this is supplemented by tyrosine converted in the body from phenylalanine. Tyrosine is a precursor to stress hormones such as dopamine, octopamine, and tyramine, and therefore plays an important role in the protective responses of insects to environmental stresses caused by, e.g., low temperatures [30]. Changes in hormone metabolism were observed in *Drosophila* species subjected to heat stress [31]. Both DOPA and dopamine are also necessary in the processes of melanisation and sclerotisation of the cuticle of insects and influence the construction of the exoskeletons during the insect’s development [32]. The increase in the proline level observed in the body of wintering aphids is probably related to its function in the processes of acclimatisation of insects to low temperatures and response to temperature stress. It has been shown that proline performs the function of stabilizing membranes and proteins [33] and is also correlated with the level of stress hormones during temperature stress [34]. Accumulation of proline may also be related to its use as an energy substrate to maintain ATP level in insect metabolism, especially when fat reserves are reduced [12]. Accumulation of proline during thermal stress could be linked also to the regulation of the pentose phosphate pathway [35]. Earlier studies also indicated the role of proline in the stabilisation of enzymes and saving their activity at cold temperature [36]. An increase in the level of proline during cold stress was also observed in *Alphitobius diaperinus, Cryptolestes ferrugineus*, and *Sitophilus granarius* [11,12]. An increase in proline, isoleucine, and valine was also observed in wintering *Kermania pistacilla* larvae, where the total amount of free amino acids was highest in November and then gradually decreased [37]. The increase in proline in the aphid body that we observed was probably due to its function as energy substrate and cryoprotectant, as in other overwintering insects [4,38]. Because the proline is synthesised from glutamate [39], the level of this amino acid was increased in aphids under cold stress, as opposed to L-5-oxoproline, where the level was higher in summer specimens, and next was converted to glutamate in winter. Moreover, glutamate plays the role as precursor for GABA (4-aminobutyric acid), an important neurotransmitter in the central nervous system. The metabolic response of aphids to low temperature, in the form of changes in the composition of amino acids, especially an increase in the levels of tyrosine and proline, suggests their use in stress protection and energy production processes.

Lipids such as unsaturated fatty acids play an important role in the processes of adaptation of insects to lower temperatures, the function of which is to maintain the stabilisation of cell membranes [40]. Increased levels of linoleic acid were observed in *Pyrrhocoris apterus*, while in *Ostrinia nubilalis*, palmitoleic and oleic acids were found. Increased levels of oleic acid were also found in the bodies of the aphids we studied, which suggests that it protects the cell membranes of the aphids from changing from a liquid to a solid state [41,42]. Oleic acid was also elevated in cell membranes during rapid cold-hardening and diapause in the *Sarcophaga crassipalpis* [43].

Lipids may also be used by insects as a source of energy during the suppressed metabolism under cold conditions. Many insects prepare for wintering through a strategy of storing energy in the form of lipids, most often in the form of triglycerides [44]. The insects can synthesise saturated and monounsaturated fatty acids from plant sterols. Fatty acids are mainly stored as triacylglycerols (TAGs) in fat body cells. An increase in the accumulation of lipid stores was also observed in diapause insects or overwintering stages of insects. The lack of accumulation of cryoprotectants and fatty acids in *K. pistilla* indicates that this insect maintains the stability of biochemical composition, which means that it goes into a quiescent state, not diapause, and uses mainly the glycogen to fuel energy metabolism [37]. Most overwintering insects end the winter with lower lipids than at the beginning, and this suggests that lipids are an important source of overwintering fuel. During the winter, the metabolism fuel mainly with lipids is prominent not only among insects belonging to Diptera and Lepidoptera, but also the Hemiptera orders [45]. Previous studies showed that the total amount of lipids in the body of *C. tujafilina* was significantly higher under cold conditions [16]. It was found that the rate of lipid consumption by overwintering insects depends on the mean and the variability of the temperature of microhabitat [44]. Wintering insects consume fatty acids mainly through the β-oxidation process. They can also carry out ketogenesis from fatty acids as observed, e.g., in overwintering *Epiblema scudderiana* moths [46].

Aphids’ symbionts play an important role in responding to environmental stress. In aphids, there are two categories of symbionts: obligate and facultative endosymbionts. The presence of obligate endosymbionts in aphids is related to their diet because aphids’ diet is deficient in many nutrients, principally amino acids, which cannot be synthesised by them and must be provided by their endosymbiont, such as *Buchnera aphidicola*. Facultative symbionts could affect the metabolism of the host, participating in host specialisation; rescuing the host from heat damage; and, last but not least, providing defence against natural enemies [47,48]. It has been shown that a lack of symbionts in *Aphis fabae* results in high aphid mortality and a low level of amino acid digestibility from the diet, which results in poor insect growth [49].

In response to the stress caused by the low temperature in the aphids’ bodies, we found an increase in shikimate acid. The presence of this acid is probably related to the symbiotic organisms that are present in the bodies of aphids and are involved in phenyloalanine, tyrosine, and tryptophan biosynthesis [47]. The presence of shikimate acid has been found in pea aphids (*Acyrthosiphon pisum*) associated with *Serratia symbiotica* [47]. Facultative symbionts could increase the levels of *N*-acetyl-D-mannosamine, a precursor to sialic acid, which is a metabolite that protects against temperature stress produced by *S. symbiotica* [47]. An elevated level of this compound was also found in the body of *C. tujafilina* under cold stress, which suggests its protective function. In amino acid biosysthesis, homoserine was also involved, the increase of which suggests synthesis of threonine and izoleucine in an overwintering aphid’s body. During cold stress, the insects also activated pathways related to oxidative stress. This was related to the emergence of reactive oxygen species (ROS), which can negatively affect insect cells. The ongoing process of oxidative stress may be indicated by an increase in the level of 2-ketoglucose dimethyl acetal, which is formed due to the addition of hydroxyl radicals [50].

On the basis of the analysis of developmental parameters, we found that the overwintering generations of *C. tujafilina* differ significantly from the summer generations, mainly due to the lower average fecundity of females, increased longevity, decreased rate of reproduction, and shortened reproductive period. However, it was shown that the biological parameters of this species do not differ significantly from the generations observed in spring and autumn on the above-ground parts of the plant [15,51]. This confirms that this species adapts to winter conditions and can develop in winter thanks to the physiological and metabolic changes in the body of the aphids.

## 5. Conclusions

Our research is the first to show the metabolic response of aphids in order to adapt to the low temperatures of winter. We showed that the metabolic profile of aphid bodies differed in summer and winter. The metabolic changes were mainly related to carbohydrate metabolism pathways such as glycolysis and the pentose phosphate pathway, a decrease in activity of TCA cycle, an accumulation of polyols, increased levels of proline and tyrosine, and increased levels of unsaturated and saturated fatty acids. By reducing their metabolic rate, aphids gain an extension to their survival time, thanks to the slow catabolism of stored lipids.

## Data Availability

The data presented in this study are available in the article. Additional data are available on request from the data curator.
